# Clinical trials in amyotrophic lateral sclerosis: a systematic review and perspective

**DOI:** 10.1093/braincomms/fcab242

**Published:** 2021-10-23

**Authors:** Charis Wong, Maria Stavrou, Elizabeth Elliott, Jenna M Gregory, Nigel Leigh, Ashwin A Pinto, Timothy L Williams, Jeremy Chataway, Robert Swingler, Mahesh K B Parmar, Nigel Stallard, Christopher J Weir, Richard A Parker, Amina Chaouch, Hisham Hamdalla, John Ealing, George Gorrie, Ian Morrison, Callum Duncan, Peter Connelly, Francisco Javier Carod-Artal, Richard Davenport, Pablo Garcia Reitboeck, Aleksandar Radunovic, Venkataramanan Srinivasan, Jenny Preston, Arpan R Mehta, Danielle Leighton, Stella Glasmacher, Emily Beswick, Jill Williamson, Amy Stenson, Christine Weaver, Judith Newton, Dawn Lyle, Rachel Dakin, Malcolm Macleod, Suvankar Pal, Siddharthan Chandran

**Affiliations:** 1 Centre for Clinical Brain Sciences, Chancellor's Building, 49 Little France Crescent, The University of Edinburgh, Edinburgh, EH16 4SB, UK; 2 Anne Rowling Regenerative Neurology Clinic, Chancellor's Building, 49 Little France Crescent, The University of Edinburgh, Edinburgh, EH16 4SB, UK; 3 Euan MacDonald Centre for MND Research, University of Edinburgh, FU303F, Chancellor’s Building, 49 Little France Crescent, Edinburgh EH16 4SB, UK; 4 UK Dementia Research Institute, Chancellor’s Building, The University of Edinburgh, 49 Little France Crescent, Edinburgh EH16 4SB, UK; 5 Department of Neuroscience, Brighton and Sussex Medical School, University of Sussex, Brighton, BN1 9PX, UK; 6 Neurology Department, Wessex Neurosciences Centre, Southampton General Hospital, Southampton, SO16 6YD, UK; 7 Department of Neurology, Royal Victoria Infirmary, Newcastle upon Tyne NE1 4LP, UK; 8 Queen Square Multiple Sclerosis Centre, Department of Neuroinflammation, UCL Queen Square Institute of Neurology, Faculty of Brain Sciences, University College London, London WC1B 5EH, UK; 9 National Institute for Health Research, University College London Hospitals, Biomedical Research Centre, London, W1T 7DN, UK; 10 MRC CTU at UCL, Institute of Clinical Trials and Methodology, University College London, London, WC1V 6LJ, UK; 11 Statistics and Epidemiology, Division of Health Sciences, Warwick Medical School, University of Warwick, Coventry, CV4 7AL, UK; 12 Edinburgh Clinical Trials Unit, Usher Institute, Level 2, NINE Edinburgh BioQuarter, 9 Little France Road, Edinburgh EH16 4UX, UK; 13 Motor Neurone Disease Care Centre, Manchester Centre for Clinical Neurosciences, Salford, M6 8HD, UK; 14 Department of Neurology, Institute of Neurological Sciences, Queen Elizabeth University Hospital, NHS Greater Glasgow and Clyde, Glasgow, G51 4TF, UK; 15 Department of Neurology, NHS Tayside, Dundee, DD2 1UB, UK; 16 Department of Neurology, Aberdeen Royal Infirmary, Aberdeen, AB25 2ZN, UK; 17 NHS Research Scotland Neuroprogressive Disorders and Dementia Network, Ninewells Hospital, Dundee, DD1 9SY, UK; 18 Department of Neurology, NHS Highland, Inverness, IV2 3UJ, UK; 19 Department of Clinical Neurosciences, NHS Lothian, Edinburgh, EH16 4SA, UK; 20 Atkinson Morley Regional Neurosciences Centre, St. George's University Hospitals NHS Foundation Trust, London SW17 0QT, UK; 21 Barts MND Centre, Barts Health NHS Trust, London, E1 1FR, UK; 22 Department of Neurology, Queen Elizabeth Hospital Birmingham, Birmingham, B15 2GW, UK; 23 Department of Neurology, NHS Ayrshire & Arran, KA12 8SS, UK

**Keywords:** amyotrophic lateral sclerosis, clinical trials, systematic review, methodology, perspective

## Abstract

Amyotrophic lateral sclerosis is a progressive and devastating neurodegenerative disease. Despite decades of clinical trials, effective disease-modifying drugs remain scarce. To understand the challenges of trial design and delivery, we performed a systematic review of Phase II, Phase II/III and Phase III amyotrophic lateral sclerosis clinical drug trials on trial registries and PubMed between 2008 and 2019. We identified 125 trials, investigating 76 drugs and recruiting more than 15 000 people with amyotrophic lateral sclerosis. About 90% of trials used traditional fixed designs. The limitations in understanding of disease biology, outcome measures, resources and barriers to trial participation in a rapidly progressive, disabling and heterogenous disease hindered timely and definitive evaluation of drugs in two-arm trials. Innovative trial designs, especially adaptive platform trials may offer significant efficiency gains to this end. We propose a flexible and scalable multi-arm, multi-stage trial platform where opportunities to participate in a clinical trial can become the default for people with amyotrophic lateral sclerosis.

## Teaser

Wong et al. reviewed historical approaches to clinical trials in amyotrophic lateral sclerosis. Lack of sensitive outcome measures, limitations in resources and barriers to trial participation were challenges for timely and definitive evaluation of drugs in two-arm trials. They concluded that future trials should be more flexible, scalable and efficient.

## Graphical Abstract

**Graphical Abstract:**
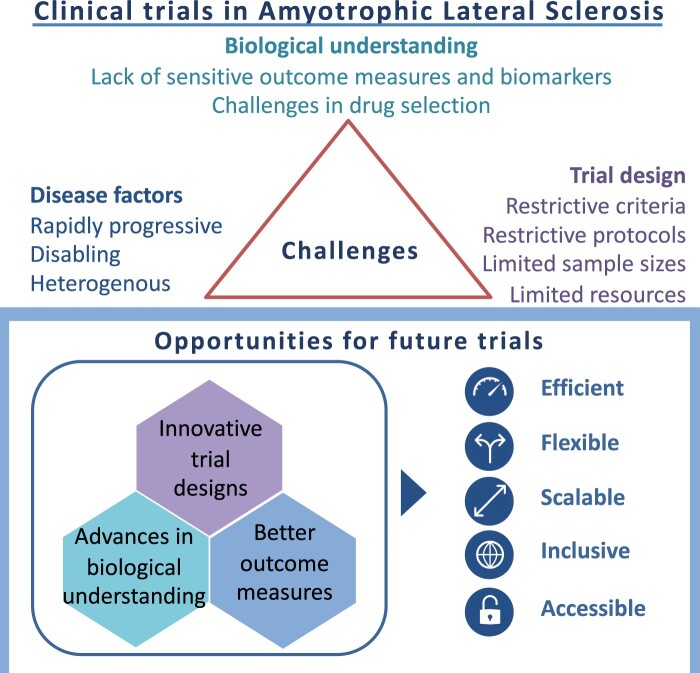

## Introduction

Amyotrophic lateral sclerosis is a rapidly progressive disease with a median survival of 2–3 years.^1^ While there has been some progress in non-pharmacological interventions, such as non-invasive ventilation and gastrostomy, and symptomatic pharmacological treatments in amyotrophic lateral sclerosis,^2^ trials in the last 25 years have largely failed to identify effective disease-modifying drugs. Riluzole, approved in 1995, is the only globally licenced disease-modifying drug and prolongs survival by just 2–3 months on average.^3^ Edaravone has been approved in some countries including USA, Canada, Japan and South Korea following a positive trial in a highly selected population.^4^ sodium phenylbutyrate-taursodiol (AMX0035), masitinib and methylcobalamin have recently emerged as promising candidates following trials in subsets of people with amyotrophic lateral sclerosis (pwALS).^5–8^ However, evidence for generalizable and substantial effects on survival for edaravone, AMX0035, masitinib and methylcobalamin is limited.

Limitations of pre-clinical models and incomplete knowledge of the underlying biology of amyotrophic lateral sclerosis are some of the factors hindering translational success.^9^ However, the last decade has seen substantial progress in our understanding of the genetic and molecular pathobiology of amyotrophic lateral sclerosis and related disorders. This improved mechanistic insight has, in turn, led to the identification of many promising therapeutic targets that justify not only further experimental study but also, in many instances, formal clinical trials. Given that at present only a small fraction of pwALS participate in clinical trials,^10^ despite a prognosis similar to that for many cancers where trial participation rates are considerably higher,^11^ there is an opportunity to learn from innovations in cancer medicine. This includes re-thinking historical approaches to drug selection and evaluation in standalone two-arm trials.

Here, we evaluate previous trial designs and consider specific methodological challenges. These include the rapid pace of decline and accumulating disability in amyotrophic lateral sclerosis, the clinically heterogeneous nature of amyotrophic lateral sclerosis and a range of trial design and conduct issues. We review emerging innovative trial designs and propose a flexible multi-arm, multi-stage trial platform model that can incorporate new candidate drugs as they are identified.

## Materials and methods

We conducted a systematic search of trial registries including clinicaltrials.gov,^12^ World Health Organization International Clinical Trials Registry Platform (ICTRP),^13^ European Union Clinical Trials Register (EU CTR),^14^ and PubMed on 9 April 2019 to identify Phase II, Phase II/III and Phase III Clinical Trials of an Investigational Medicinal Product (CTIMPs) assessing potential disease-modifying drugs in amyotrophic lateral sclerosis. We searched clinicaltrials.gov for all interventional trials of ‘amyotrophic lateral sclerosis’ or ‘motor neuron disease’. We searched ICTRP and EU CTR for interventional trials of ‘amyotrophic lateral sclerosis’ using ‘Phase II’ and ‘Phase III’ filters. We searched PubMed for ‘(“motor neuron disease” OR “amyotrophic lateral sclerosis”) AND (Clinical Trial [ptyp])’. Trials registered, completed or published between 1 January 2008 and 9 April 2019, excluding extension trials, were included. No language restrictions were applied.

### Statistical analysis

We performed a narrative synthesis on all included trials to summarize trials according to phase, study status, study duration, number of participants, investigational medicinal products (IMPs), eligibility criteria and primary outcome measures. For studies with entries on multiple registries, data from ClinicalTrials.gov were used as the primary data source. For studies registered on registries with results published in journals, publications were used as primary source of data. To review sample size considerations including recruitment and retention, we summarized trials for completed trials with efficacy-based primary outcome measures according to number of study arms, number of participants recruited, number of participants recruited per arm, withdrawal rates and reasons for withdrawal.

### Data availability statement

Data for this review are available in the Supplementary material.

## Results

### Characteristics of trials

**Figure 1:**
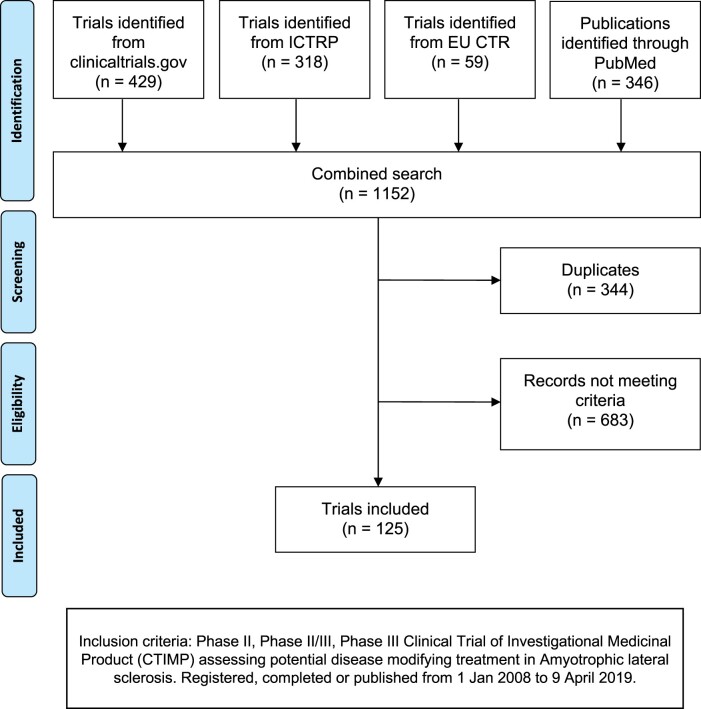
PRISMA diagram of systematic review.

A total of 1152 records were identified [see Preferred Reporting Items for Systematic Reviews and Meta-Analyses (PRISMA) diagram in Fig. 1]: 344 were duplicates, and 683 were excluded because they did not meet our eligibility criteria. Total of 58 records on PubMed corresponded with trials identified from the registry search. In total, 125 trials, with a total of 15 647 participants, were identified and included in the analysis. Characteristics of these trials are summarized in Table 1. A total of 73 trials were recorded as completed. Only 6 of the 125 trials were terminated prior to planned completion: 2 of these were trials with novel designs terminated for futility^15^^,^^16^; 1 was terminated due to adverse events^17^; 1 was terminated after a participant had rapid progression^18^; the reasons for termination for two studies were not stated. Trial duration ranged between 4 and 95 months (median 25 months).

**Table 1:** Phase II, Phase II/III and Phase III CTIMP assessing potential disease*-*modifying treatment in amyotrophic lateral sclerosis registered, completed or published from 1 January 2008 onwards excluding extension trials according to status, phase and trial design

Description	Number of trials
**By status**	
Active	35
Completed, has results	61
Completed, no results	12
Not yet recruiting	5
Terminated	6
Unknown	4
Withdrawn	2
**Total**	**125**
**By phase**	
Phase II	97
Phase II/III	8
Phase III	20
**Total**	**125**
**By trial design**	
Non-randomized, open label	26
Randomized	96
Parallel: 87	
Crossover: 7	
Factorial: 1	
Assignment not specified: 1	
Parallel, randomization not specified	1
Not specified	2
**Total**	**125**

### Eligibility criteria

About 73% of trials specified amyotrophic lateral sclerosis disease duration within their inclusion criteria. Median permitted maximum disease duration was 36 months [interquartile range (IQR) 24–36 months]. Amyotrophic lateral sclerosis participants at King’s stage 4 (requiring enteral and/or respiratory support) were excluded from 119 trials. Total of 53 trials stipulated use of riluzole as part of their inclusion or exclusion criteria.

### IMPs

A total of 76 IMPs were evaluated in these 125 trials (Supplementary Tables 1 and 2), of which 10 IMPs were tested in 3 or more trials. The most frequently tested intervention was lithium, tested in 10 trials (6 Phase II, 2 Phase II/III and 2 Phase III), involving a total of 1414 pwALS. A total of 82 trials used oral forms of IMPs, but only 7 of these specified forms, such as liquid, soluble and powder preparations which could be delivered through enteral feeding tubes, an important consideration raised in engagement with pwALS. Two IMPs have been licenced since riluzole was approved in the USA in 1995 and the European Union (EU) in 1996. Edaravone was licenced in Japan in 2015 and in the USA in 2017 after a study showed improvement in the Amyotrophic Lateral Sclerosis Functional Rating Scale Revised (ALSFRS-R, a multi-domain functional rating scale) in a small subset of pwALS characterized by early amyotrophic lateral sclerosis, functional independence with no respiratory insufficiency and a decrease in ALSFRS-R of 1–4 points during a 12-week observation period.^4^ However, in the EU, Mitsubishi Tanabe Pharma GmBH withdrew their application for marketing authorization for edaravone after the European Medicines Agency (EMA) cited concerns on the strength of evidence for efficacy. Areas flagged included: to establish if the decrease in ALSFRS-R is considered clinically relevant in the context of evidence from other studies, generalizability of results to a European population, and the need to provide confirmatory efficacy data on survival and survival-based endpoints, such as tracheostomy and non-invasive ventilation.^19^ A previous trial with broader inclusion criteria and a trial of participants with more severe disease had not shown evidence of efficacy.^20^^,^^21^ A subsequent, retrospective single-centre analysis suggests improved survival in pwALS treated with edaravone compared with a historical control group.^22^ Conversely, edavarone did not significantly improve survival in a multicentre Italian observational study^23^ or in a surveillance evaluation by the US Department of Veterans Affairs.^24^

Masitinib in combination with riluzole reduced progression of ALSFRS-R in ‘normal progressors’ in a Phase II/III study^7^ compared to riluzole with placebo, leading to orphan drug designation being granted in Europe and the USA. However, EMA refused marketing authorization for masitinib, based on lack of reliable evidence of its benefits, citing concerns on patient stratification leading to loss of generalizability, aspects of study conduct and the handling of missing data.^25^

More recently, Food and Drug Administration (FDA) and EMA have granted orphan drug designation for AMX0035 following a positive Phase II trial^6^ in which AMX0035 reduced rate of decline of ALSFRS-R by 0.42 points/month over 24 weeks compared to placebo. Although there was no difference in survival between treatment groups during the primary analysis, subsequent analysis of long-term open-label extension results showed a significant improvement in survival [hazard ratio (HR) = 0.56, *P* = 0.023] in participants originally randomized to AMX0035 compared to participants originally randomized to placebo.^5^ However, it is notable that overall sample size was small, only 20% of participants remained in the extension trial at the time of publication, and the cohort was highly enriched using strict eligibility criteria and thus, treatment effect may not be generalizable to the wider amyotrophic lateral sclerosis population. In April 2021, FDA expressed an interest in seeing data from an additional placebo-controlled trial prior to receiving a New Drug Application for AMX0035.^26^

In a *post hoc*-analysis of a long-term Phase II/III randomized controlled study, ultra-high-dose methylcobalamin treated pwALS prolonged time to death or ventilation support and reduced decrease in ALSFRS-R score compared to placebo for pwALS diagnosed and entered into trial within 12 months of symptom duration.^8^ However, the primary analysis for the trial did not show any significant difference between treatment groups.^8^

Nuedexta (dextromethorphan/quinidine) was approved for treatment of pseudobulbar affect in amyotrophic lateral sclerosis in 2011 by the FDA and 2013 by EMA. Following reports from pwALS noting an improvement of their bulbar symptoms on Nuedexta, a Phase II randomized, blinded, crossover study of 60 participants was performed. Nuedexta was associated with significantly improvement in the Centre for Neurologic Study Bulbar Function Scale (CNS-BFS), a self-reported measure of swallowing, salivation and speech, compared to placebo over a treatment period of 28–30 days.^27^ However, it is unclear if this reduction translates into a clinically meaningful effect. Longer term larger studies are required to establish its effects on disease progression. Of note, the marketing authorization for Nuedexta has been withdrawn in the EU on request of the manufacturer due to commercial reasons.

### Trial design

About 90% of the included trials used traditional fixed designs where each trial evaluated one active IMP (including dose-ranging studies testing one active IMP at multiple doses) and were designed, conducted then analysed in a consecutive manner, with no flexibility to adopt changes, which may become desirable over the course of the trial. A total of 12 trials used ‘novel’ or ‘alternative’ trial designs (Table 2). In the Airlie House amyotrophic lateral sclerosis clinical trial guidelines,^28^ such designs include adaptive studies, seamless Phase II/III designs, enrichment designs and futility designs.

**Table 2:** Novel trial designs for Phase II, Phase II/III and Phase III CTIMPs evaluating potential disease*-*modifying treatment of amyotrophic lateral sclerosis

Novel trial designs	Completed with results	Conclusive results on efficacy/ futility	Early stopping due to predefined futility criteria	Number of trials
Multi-arm studies^a^				2
NCT01257581: Phase II study comparing Creatine 30 mg versus tamoxifen 40 mg versus tamoxifen 80 mg	Y	N	N/A	
NCT00355576: Phase II study comparing minocycline and creatine versus celecoxib and creatine	Y	N	N/A	
Multi-phase study				1
NCT00349622: Phase I-III study of ceftriaxone	Y	Y	N	
Sequential/adaptive design				6
NTR 1448: Phase II study of lithium	Y	Y	Y	
NCT00136110: Phase III study of sodium valproate	Y	Y	Y	
NCT00690118: Phase II study of pioglitazone	Y	Y	Y	
NCT00243932: Phase II study of coenzyme Q10	Y	Y	Y	
NCT00818389: Phase II/III study of lithium in combination with riluzole	Y	Y	Y	
NCT02238626: Ibudilast (MN-166) in subjects with amyotrophic lateral sclerosis (IBU-ALS-1201)	N	N/A	N/A	
Phase II futility design				1
EudraCT 2014–005367-32: Phase II study of guanabenz	N	N/A	N/A	
Historical controls, primarily virtual data collection				1
NCT02709330: amyotrophic lateral sclerosis reversals—Lunasin regimen	Y	Y	N/A	
Adaptive seamless Phase II/III				1
NCT00706147: Phase II/III randomized, Placebo-controlled trial of arimoclomol in SOD1 positive familial amyotrophic lateral sclerosis	Y (closed after Phase II due to slow recruitment)	N	N	
Total	10	7/10 (70%)	5/7 (71%)	12

a For multi-arm studies, trials specified evaluated more than one active investigational medicinal product; studies including concomitant use of riluzole and dose-ranging studies were not included here.

**Figure 2:**
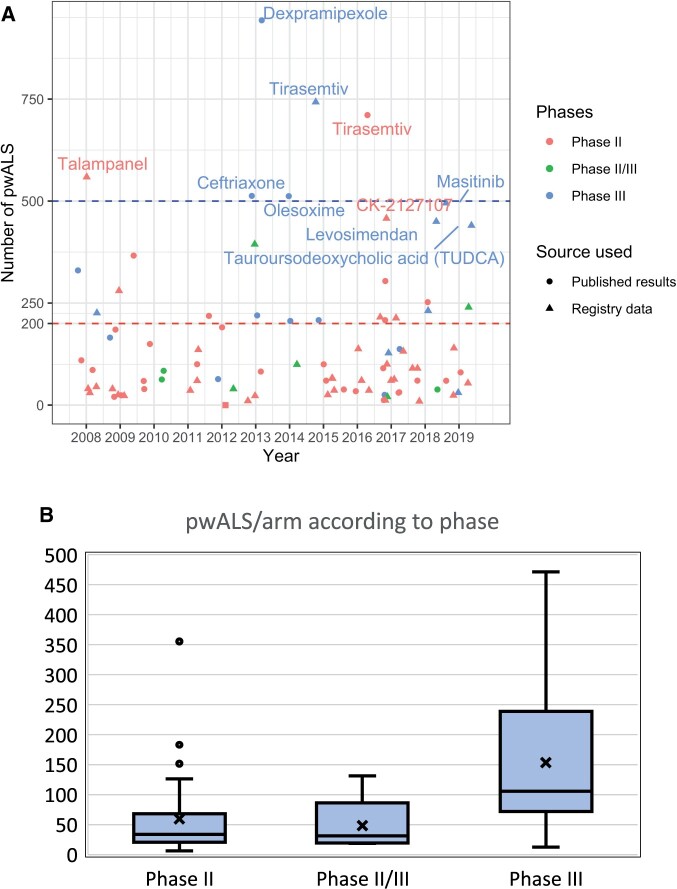
Number of pwALS participating in trials. (**A**) (top) shows number of pwALS recruited per trial by phase and year for trials with efficacy-based outcome measures. Published results were used as the source where available (denoted by circle), and registry data were unavailable (denoted by triangles). The 10 trials with highest number of pwALS/trial are labelled with the intervention tested—these trials were two-arm trials with the exception of the following dose-ranging trials: tirasemtiv Phase III (4-arm), masitinib (3-arm), CK-2127107 (4-arm) and talampanel (3-arm). The dashed horizontal lines reflect our sample size calculations using PROACT data: the red line indicates *n* = 200 (100/arm) required for a 2-arm RCT with randomization of 1:1, powered at 85% to detect a 25% difference in rate of ALSFRS-R decline over 12 months with a test conducted with a two-sided 20% significance level. The blue line indicates *n* = 500 (250/arm) required to evaluate one IMP in a two-arm RCT with randomization of 1:1, powered of 90% to detect a HR of 0.65 for survival over 2 years with a two-sided 5% significance level. (**B**) (bottom) shows a box and whiskers plot of the number of pwALS recruited per study arm according to CTIMP phase for Phase II (32 trials), Phase II/III (5 trials) and Phase III (12 trials) CTIMPs evaluating potential disease-modifying treatment of amyotrophic lateral sclerosis.

Next, we reviewed sample size considerations including recruitment and retention. To do this, we analysed studies with efficacy-based primary outcome measures (Table 3). Results were available for 49 (32 Phase II, 5 Phase II/III and 12 Phase III) of the 89 trials with efficacy-based primary outcome measures. A total of 37 studies evaluated two arms, 5 were single-arm (including 1 using historical controls^29^), 6 trials evaluated three arms (5 studies of one IMP each at different doses and 1 multi-arm trial of two active IMPs) and a single trial had four arms (testing one IMP at three doses against placebo). The total number of participants recruited per trial ranged from 23 to 943. The median number of participants per arm was 43 (IQR 23–107) (Fig. 2). Only 26 of 49 trials (20 Phase II, 4 Phase II/III, 2 Phase III) recruited fewer than 100 participants in total, and only 4 Phase III trials recruited more than 500 participants. Excluding trials with adaptive designs, which were terminated early, 87% of the studies achieved at least 90% of their target enrolment. However, attrition significantly affected studies: 40% of trials had an attrition rate (calculated as the proportion of recruited patients who did not complete studies for reasons other than death) of more than 20% (range 0–70%, median 16%). In these studies, median percentage of participants withdrawing due to adverse events was 26% (IQR 0–45%, range 0–100%). Other reasons for withdrawal included participant request, protocol violation, loss to follow up, perceived lack of efficacy, non-compliance, disease progression felt to be due to study drug, burden of participating, physician or investigator’s decision and reaching protocol-defined stopping point other than death.

**Table 3:** Phase II, Phase II/III and Phase III CTIMP assessing potential disease-modifying treatment in amyotrophic lateral sclerosis registered, completed or published from 1 January 2008 onwards excluding extension trials according to type of primary outcome measure

Type of primary outcome measure	Number of trials			
	Phase II	Phase II/III	Phase III	Total
Efficacy	**63**	**8**	**18**	**89**
Functional rating scales	**39**	**4**	**8**	**51**
ALSFRS-R	36	4	7	47
AALSRS	1	0	1	2
CNS-BFS	1	0	0	1
Penetration aspiration scale	1	0	0	1
Combined survival/functional outcome measure e.g. Joint rank scales/CAFS	**1**	**1**	**2**	**4**
Staging	**3**	**1**	**0**	**4**
ALS-MITOS	1	0	0	1
Loss of self-sufficiency	1	0	0	1
Time to tracheostomy/death	0	1	0	1
Not specified	1	0	0	1
Survival	***8***	***1***	***6***	***15***
Respiratory function (SVC)	***2***	***0***	***1***	***3***
Muscle strength	***3***	***1***	***1***	***5***
Manual muscle testing (MMT)	1	1	1	3
Isometric arm strength	2	0	0	2
Biomarkers	***7***	***0***	***0***	***7***
Cerebrospinal fluid	2	0	0	2
Blood	1	0	0	1
Neuroradiology	1	0	0	1
Neurophysiology	3	0	0	3
Pharmacodynamics/pharmacokinetics	**4**	**0**	**0**	**4**
Safety and tolerability	**30**	**0**	**2**	**32**

AALSRS = Appel amyotrophic lateral sclerosis rating scale; ALSFRS-R = amyotrophic lateral sclerosis functional rating scale revised; ALS-MITOS = amyotrophic lateral sclerosis Milano–Torino staging; CAFS = combined assessment of function and survival; CNS-BFS = Centre of Neurologic Study Bulbar Function Scale; MMT = manual muscle testing; SVC = slow vital capacity.

### Outcome measures

Total 24 of the 49 studies used ALSFRS-R, while 10 trials used survival, and 2 used compound scores combining survival and ALSFRS-R as primary outcome measures.^30^^,^^31^ Other primary outcome measures included alternative functional rating scales, muscle strength testing, disease staging, respiratory function and biofluid biomarkers (Table 3). The substantial variation in primary endpoints used reflects the lack of consensus on what is considered a relevant and clinically important improvement for confirmatory trials, and the lack of sensitive and reliable outcome measures to detect potential treatment effects in exploratory trials.

ALSFRS-R scores were reported as mean rate of decline or slope of decline, or as an absolute or percentage change from baseline score, measured over durations ranging between 8 and 90 weeks. Trials using ALSFRS-R were planned to detect a range of treatment effects, including a reduction in the rate of decline of anything from 10% to 50%, or a 3-point difference in ALSFRS-R scores over 24–40 weeks.

Survival results were reported in several ways including number of deaths, proportion alive at a specified timepoint, Kaplan–Meier curves, time-to-event including death or proxies for survival such as tracheostomy or the initiation of permanent assisted ventilation. Follow-up periods for survival ranged from 12 to 18 months.

Biomarkers were specified as outcome measures in 42 (34%) of trials: 27 trials included biofluid biomarkers, 3 measured imaging biomarkers and 13 measured neurophysiological biomarkers.

### Proportion of pwALS in clinical trials

To determine the approximate proportion of pwALS participating in clinical trials, we evaluated reported recruitment data for trials identified in our systematic search and compared this to amyotrophic lateral sclerosis epidemiology data (Supplementary Table 3). We identified 66 studies, which recruited in the UK and the USA and extracted the total number of participants from these countries. This was based on published data where these were available; and where not, by taking the proportion of recruiting centres in the UK or USA multiplied by the total number of participants. Applying this approach to those studies where the country of recruitment was known gave estimated recruitment within 1% (2887 versus 2916 observed) of the true value. We calculated trial participation rates using an estimated annual incidence of amyotrophic lateral sclerosis of 2/100 000^32^ and population data from national statistics. We estimate that fewer than 5% of pwALS in the UK and 8% of pwALS in the USA participated in a CTIMP during this period. Globally, we estimate that the 15 647 pwALS who participated in these 125 CTIMPs between 2008 and 2019 represent around 2.5% of the total number of new cases of amyotrophic lateral sclerosis during this period.

## Discussion

Our analysis of trials between 2008 and 2019 found 76 IMPs evaluated in 125 trials, predominantly with two-arm trial designs with a range of sample sizes and a variety of outcome measures. We estimate that fewer than 5% of newly diagnosed pwALS entered a clinical trial. Limitations of this review include incomplete reporting of completed trials, which may reflect reporting bias. A total of 73 trials were recorded as completed, but for 12, the results were not available in a manner consistent with EU or FDA regulations. Of these 12, 2 did not provide completion dates, 2 were completed within the preceding 12 months and 8 were completed more than 12 months prior, noting that FDA and EU regulations mandate reporting of results within 12 months of completion. Three of these trials published results in either journals or on trial registries between April 2019 at the time of writing,^7^^,^^33^^,^^34^ while three had published conference proceedings but these did not contain the full dataset required by regulations. Furthermore, there was inconsistency of data across data sources for a few trials, where conflicting data were recorded on different trial registries or in trial publications. For our review, we analysed data from the primary source as specified in our methods. Ideally, we would group studies according to their aims for our analysis. We would divide studies into exploratory versus confirmatory studies and subdivide exploratory studies according to their main aims including safety, dose-ranging or efficacy. However, most trial registries do not currently include such information. Thus, we limited our analysis to Phase II and Phase III studies, using the primary outcome measure presented to infer the primary aim of each study.

Here, we discuss challenges in designing, delivering and conducting trials in amyotrophic lateral sclerosis, and review the possible causes of low proportion of pwALS entering CTIMPs.

### Understanding of disease biology

While our understanding of amyotrophic lateral sclerosis disease biology, especially in genetics and molecular biology, has improved over recent years, there remains significant gaps in our knowledge of mechanistic targets and networks in amyotrophic lateral sclerosis.^9^ This impedes improvements in disease models, drug development and selection for clinical trials.^9^^,^^35^ Furthermore, the limitations in disease understanding also poses challenges in developing more reliable, objective, sensitive and specific biomarkers of disease progression and prognostication. Notwithstanding recent advances in biomarker development, these are yet to be established and validated as primary efficacy-based outcome measures in clinical trials.^36^ Thus, we continue to rely on clinical rating scales and survival to evaluate efficacy and narrow inclusion criteria to reduce heterogeneity in trial cohorts. This, in turn, has implications on sample sizes, trial duration, access to trials and generalizability of results as discussed further below.

### Disease-related factors

Heterogeneity of clinical presentation, site of onset and rate of progression often result in diagnostic delay, with an average time from symptom onset to diagnosis of 12–15 months.^37^ Combined with the multiple manifestations, rapidly progressing, disabling and short survival of the disease, this results in a short-time window to identify, screen and recruit pwALS. Furthermore, adherence with trial protocols often becomes challenging with increasing disability; movement, ambulation, speech, swallowing and respiration are commonly affected, the latter causing some pwALS to become dependent on enteral feeding and ventilatory support. Data from Clinical Audit Research Evaluation for Motor Neurone Disease (CARE-MND),^38^ the highly curated Scottish MND register, showed that a quarter of pwALS in Scotland between 2015 and 2019 had a gastrostomy and 17% used non-invasive ventilation. This is particularly problematic for trials where invasive procedures, such as intrathecal delivery of IMPs or lumbar punctures, or maintained ability to swallow oral medications are required. Distant travel to trial centres to attend face-to-face appointments is likely to be physically demanding and burdensome for pwALS with accumulating disability. Cognitive and behavioural changes are well recognized and worsen with disease progression,^39–41^ forming another barrier to trial participation, including issues around informed consent, and retention.

### Narrow versus broad trial inclusion criteria

In addition to practical and logistical considerations, phenotypic heterogeneity in amyotrophic lateral sclerosis adds complexity to participant selection. This includes variation in survival, which is well recognized in amyotrophic lateral sclerosis.^42–45^ In a Scottish cohort (2015–16), ∼10% pwALS are long survivors, defined as survival beyond 8 years, with median survival from onset of 15.6 years.^42^ Other sources of heterogeneity with prognostic implications include age at onset, site of presentation, respiratory involvement, subtypes of amyotrophic lateral sclerosis, genetic heterogeneity and diagnostic certainty.^1^

To create a more homogenous cohort, increase trial protocol adherence and exclude long survivors, investigators often use restricted eligibility criteria as demonstrated in our review as well as a review of amyotrophic lateral sclerosis clinical trials between 2000 and 2017 by van Eijk et al.^10^ However, employing strict eligibility criteria inevitably affects the generalizability of results. Furthermore, it reduces the proportion of eligible participants in a condition where prognosis is grave and treatment options are severely limited. Van Eijk et al.^10^ found that on average 60% of pwALS were ineligible to participate in clinical trials, with more than 20% of pwALS excluded because of El Escorial criteria stipulations alone. Along with restrictions of symptom/disease duration, this has particular implications on recruitment of patients of certain phenotypes,^46^ such as progressive bulbar palsy, flail arm or flail leg syndrome, primary lateral sclerosis and progressive muscular atrophy. Notably, the complexity and poor test-retest reliability of the revised El Escorial criteria has been highlighted in a recent study,^47^ leading to the proposal of the Gold Coast criteria^48^ where diagnostic criteria for amyotrophic lateral sclerosis is simplified and the categories of possible, probable and definite amyotrophic lateral sclerosis in the El Escorial criteria collapsed into a single entity. In our review, 95% of trials excluded pwALS who were enterally or ventilator supported while 42% either mandate riluzole co-treatment or exclude pwALS on concomitant riluzole. Of note, 45% pwALS in Scotland were started on riluzole between 2015 and 2020, 15% of whom subsequently discontinued riluzole.^49^ Indeed when applying the eligibility criteria of the edaravone 2017 trial^4^ to an incident cohort, only 10% pwALS would have been eligible.^10^ Nevertheless, the authors found that strict eligibility criteria only minimally reduced heterogeneity in trial endpoints.^10^

An alternative approach is to adopt broad inclusion criteria recruiting all subtypes of amyotrophic lateral sclerosis including long survivors, supported by protocol innovations to allow participation of pwALS with more advanced disease. Examples include liquid IMP preparations and home/telephone/video assessments.^50^ However, this approach requires larger sample sizes to enable a sufficiently powered trial to account for a potentially reduced effect size in a more heterogeneous cohort, or a prospective analysis plan that excludes ALSFRS-R (but not survival) data, from participants who are beyond 8 years of disease onset at trial entry.

The recent development of personalized risk prediction models,^1^^,^^45^ based on clinical and genetic data, including the presence of a ‘C9orf72’ repeat expansion, could be used for stratification in future trials and might achieve a better balance between endpoint heterogeneity and exclusion rates. Van Eijk et al.^10^ calculated that incorporating the European Network for the Cure of Amyotrophic Lateral Sclerosis risk model^1^ for participant selection in the edaravone 2017 trial^4^ could increase proportion of pwALS eligible nearly fivefold while maintaining similar power.

### Designing clinical trials to evaluate clinically meaningful change

The tension between practical considerations of feasibility of recruitment, compliance and retention, versus adequate power for evaluation of meaningful outcome measures is apparent throughout the amyotrophic lateral sclerosis trial literature. Indeed, what constitutes a clinically meaningful change on these endpoints is difficult to define. The range of primary outcome measures and primary endpoints reflects the absence of widely available, robust and sensitive biomarkers. There is, however, an encouraging trend towards the use and development of biomarkers in more recent trials. Out of 29 trials including biomarkers as outcome measures with trial start dates available in registries, 21 (72%) were conducted within the past 5 years. Meanwhile, there remains a dependency on clinical ordinal scales, in particular the ALSFRS-R. ALSFRS-R was the most common primary outcome measure used (41% of Phase II studies and 35% of Phase III studies). Survival was the second most common primary outcome measure used in Phase III studies (30%).

**Figure 3:**
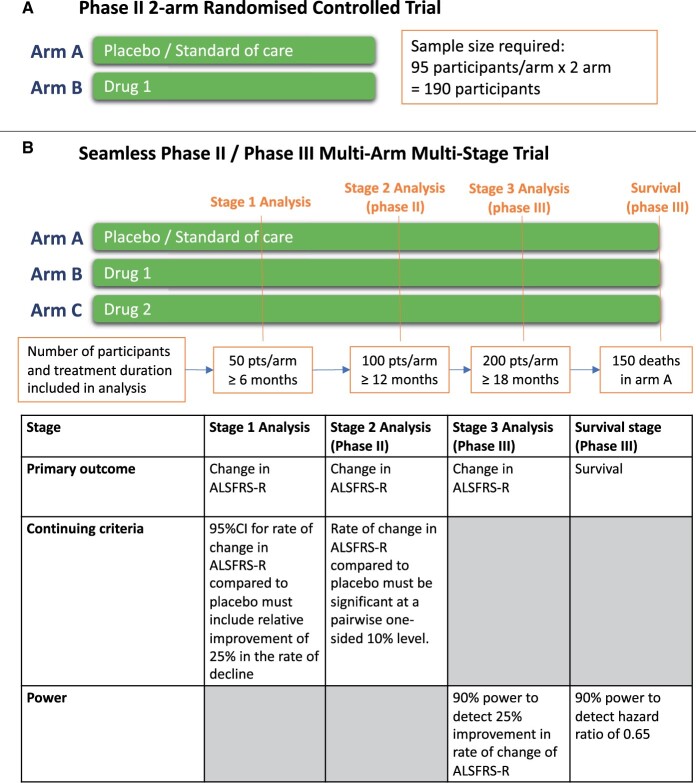
Example sample size requirements according to trial designs. (**A**): traditional 2-arm Phase II trial with randomization of 1:1 to evaluate 1 IMP against placebo or control with sample size calculated based on 85% power for a 20% two-sided significance level test to detect a 25% difference in rate of ALSFRS-R decline at 12 months calculated using data from PROACT.^56^ (**B**): a seamless Phase II/Phase III multi-arm multi-stage trial with randomization of 1:1:1 with interim analysis where arms can be stopped if predefined efficacy and safety criteria are not met.

Although ALSFRS-R is widely used and validated, a Rasch analysis identified that ALSFRS-R total score lacks unidimensionality, meaning investigators cannot be confident that the same total ALSFRS-R score in two pwALS reflects comparable clinical conditions.^51^^,^^52^ The use of total ALSFRS-R score is particularly problematic where differences in treatment responses between subscales increase and may dilute treatment effects.^53^ Furthermore, as ALSFRS-R plateaus and small reversals are common, stable ALSFRS-R scores over short intervals may not represent treatment effect and has implications for trial duration.^54^ Notwithstanding these issues, the sample size required to detect a clinically meaningful change in ALSFRS-R^55^ is considerable. Using simulation models based on the Pooled Resources Open-Access Amyotrophic Lateral Sclerosis Clinical Trials (PROACT) database,^56^ we calculate that ∼200 participants (100/arm) are required for a two-arm randomized controlled trial (RCT) with randomization of 1:1, to provide 85% power to detect a 25% difference in rate of ALSFRS-R decline over 12 months with a test conducted with a two-sided 20% significance level (Fig. 3A). We believe this statistically liberal approach is appropriate in Phase II. Similarly, noting that survival is considered the gold-standard outcome for Phase III and using natural history data obtained from CARE-MND,^38^ we estimate that >500 patients are required to evaluate one IMP in a two-arm RCT with randomization of 1:1 and 2 years follow up, to achieve a power of 90% to detect a HR for death of 0.65 with a two-sided 5% significance level. A larger sample size would be required to account for the increased heterogeneity should trials use broader inclusion criteria.

These sample sizes demonstrate a major challenge in amyotrophic lateral sclerosis trials. More than half of the studies in our review with efficacy-based primary outcome measures recruited fewer than 100 participants, notwithstanding allowances for missing data, dropouts and death. Thus, with hindsight, these studies were highly unlikely to answer—in a statistically robust manner—whether the proposed intervention was clinically effective or warranted further evaluation in a Phase III trial. The inability to determine drug efficacy or futility in a single, definitive and timely trial, may lead to re-evaluation of the same IMP in subsequent trials. This is inefficient in time, cost and patient resource.

### Regulatory variation across countries

Another important challenge in amyotrophic lateral sclerosis trials is the variation in regulatory requirements and complex administrative processes across different countries and jurisdictions. These often hinder and delay multinational clinical trials as demonstrated in other diseases and may detract pharmaceutical companies from running trials in some countries.^57^^,^^58^ This is a particular challenge to conducting sufficiently powered, definitive trials in diseases like amyotrophic lateral sclerosis where prevalence is low. Furthermore, as shown in the case of edaravone, regulatory authorities vary considerably in the level and extent of evidence for efficacy considered sufficient for drug approval. This reflects one of many ethical dilemmas in amyotrophic lateral sclerosis clinical trial regulations. On one hand, regulatory bodies exist to protect and safeguard public health, and does so by ensuring that only drugs with acceptable efficacy and safety profiles are approved. On the other, pwALS have strongly advocated for access to treatments, stating that they are willing to accept greater risks that drugs are harmful or ineffective in the face of a fatal incurable illness.^26^ Indeed, more than 50 000 people signed a petition by the amyotrophic lateral sclerosis Association and I AM amyotrophic lateral sclerosis to this effect, requesting that FDA make AMX0035 available based on the Phase II trial results.^59^ Some argue that so called ‘real-world evidence’ from post-marketing observational studies can substitute for high quality evidence from large RCTs.^60^^,^^61^ While such observational studies are useful in detecting rare adverse events and (possibly) large, unexpected beneficial effects, the potential for such studies to determine moderate efficacy or safety signals accurately is limited by inherent biases.^62^ There are well established licencing pathways, such as Fast Track (FDA) or PRIority MEdicine—EMA, to accelerate evaluation of IMPs, and programmes such as expanded access (FDA), compassionate use (EMA) or Early Access to Medicines Scheme (UK), which allow access to IMPs for pwALS who are unable to access these in the context of clinical trials. One strategy, therefore, would be for trialists to increase their probability of trial success by recruiting more homogenous cohorts through narrower inclusion criteria, with pwALS who do not meet these criteria able to access these drugs via expanded access programmes. However, regulators will usually only provide a marketing authorization for the population in which the IMP has shown efficacy in RCTs. The processes through which amyotrophic lateral sclerosis stakeholders (industry, trialists, clinicians and pwALS) navigate clinical development and regulatory approval is therefore complex, and much would be gained from harmonization and increasing clarity and consistency in national trial regulations, their interpretation and implementation. The Organisation for Economic Co-operation and Development has set out stratified recommendations to this end.^63^

### Proposal for future trials

While efforts to harmonize national regulations are underway, considering the small proportion of pwALS entering interventional clinical trials and the increasing emergence of candidate medicines, there is an opportunity to improve trial design in ALS. This requires innovation in how drugs are selected and how trials are undertaken. The emerging trends in drug selection encompass four broad approaches; machine learning assisted systematic evaluation of the pre-clinical and clinical literature,^64^ phenotypic high-throughput drug screening platforms adopting human induced pluripotent stem cell modelling of brain cells,^65^^,^^66^ expert panel evaluation of candidate drugs, and more target led discovery of novel putative medicines. Moreover, the emergence of gene-based treatment approaches for monogenetic causes of amyotrophic lateral sclerosis has already led to genetically stratified treatment trials that bring additional considerations including the nature of the control arm.^67^^,^^68^ Regardless of how drugs are selected, the pace of growth in both our biological understanding of amyotrophic lateral sclerosis and linked new technologies for accelerated drug screening and discovery heralds an era where multiple biologically plausible putative medicines will be identified.

Innovative trial designs pioneered in cancer medicine^69^^,^^70^ and successfully adopted in a small number of amyotrophic lateral sclerosis trials may offer a new standard. In our review, 7 out of 10 of these trials, where results were available, delivered conclusive answers on lack of efficacy of IMPs (Table 2). Five^15^^,^^16^^,^^71–73^ out of seven trials with adaptive designs and predefined stopping criteria were able to identify futile treatments and stop trials early, reducing exposure of pwALS to ineffective treatments. An adaptive multi-phase approach in evaluating ceftriaxone enabled timely, seamless transition between phases, removing the need for multiple submissions for funding and regulatory approvals.^74^

Another class of trial design that offers promise for amyotrophic lateral sclerosis is the platform trial. These are trials, which evaluate multiple treatments simultaneously using a single master protocol, typically against a shared control or standard-of-care arm. Platform trials are especially effective where there are several promising medicines without any *a priori* evidence to favour one over the other.^75^^,^^76^ In this regard, such trials offer significant gains in efficiency by using a single master protocol as opposed to running multiple and inevitably consecutive conventional two-arm studies. Recently, such designs have enabled trial lists to identify the efficacy of dexamethasone in COVID-19 at speed.^77^ Platform trials may include adaptive features, such as in multi-arm multi-stage (MAMS) platform trials (Fig. 3B). In these, interim analyses are performed at pre-specified timepoints, with treatment arms being discontinued if they do not reach predetermined activity or safety outcomes. Seamless Phase II into III transition can also be prospectively incorporated into the trial protocol and activated if a given arm meets predefined criteria. With appropriate statistical approaches, it is possible to use outcome information gathered from participants in Phase II in the Phase III part of the trial. Further new treatments can be introduced and tested within the protocol.

The time and cost efficiencies gained by stopping ineffective arms early, while being able to introduce new arms in what is in effect a continuous trial platform without initiating further independent RCTs, are significant. It is estimated that a multi-arm study evaluating two active arms within the same trial against control halves the cost of separate traditional two-arm RCTs to evaluate two drugs.^76^ The Systemic Therapy in Advancing or Metastatic Prostate cancer: Evaluation of Drug Efficacy trial is an example of a successful MAMS trial. Total of 10 treatments have been evaluated over 20 years, resulting in change in standard-of-care three times.^78–80^ If standard two-arm RCTs had been used, it has been estimated that evaluating the same number of treatments would have required more than 40 years.^78–80^ Building on this success, similar adaptive trials have been initiated in melanoma, glioblastoma multiforme and Ebola.^81^

Notwithstanding the benefits of MAMS platform, they present significant operational and statistical challenges. One operational challenge is the need for substantial and sustained funding to enable the creation of an integrated and long-term administrative, project and statistical trial infrastructure.^82–84^ Statistical analysis plans for MAMS can be complex and include the need to calculate the type I error, or the choice of stopping boundaries, on how to adjust boundaries when the variance of a normally distributed endpoint is unknown, on the impact of adding a treatment arm during a MAMS trial or on whether additional patients should be allocated to the control group in such circumstances.^85–88^ The organization of interim analyses must also be efficient, with data collection, monitoring and statistical analysis undertaken against tight deadlines, particularly when aiming for a seamless transition from Phase II to Phase III.

Accordingly, the development of platform trials in amyotrophic lateral sclerosis is gathering momentum with three declared initiatives: USA-based HEALEY amyotrophic lateral sclerosis Platform Trial^89^ (ClinicalTrials.gov registration number NCT04297683), European-based Treatment and Research Initiative to Cure amyotrophic lateral sclerosis ‘MAGNET’ platform trial^68^ and UK-based Motor Neurone Disease—Systematic Multi-Arm Randomized Trial^90^ (ClinicalTrials.gov registration number NCT04302870). Each will have a master protocol and is multi-arm, although they vary in terms of inclusion criteria, genetic stratification, selection method of IMPs and the use of trial adaptation. Specifically, the HEALEY trial is designed to evaluate multiple drugs under a master protocol with sharing of participants on placebo across treatment regimens and frequent interim analyses to allow early stopping for success or futility of individual regimens. Participants will be randomized firstly to a treatment regimen, followed by 3:1 randomization to either active treatment or placebo. Current listed arms are zilucoplan, verdiperstat and CNM-Au8. The MAGNET trial is a Phase III adaptive platform trial, which will use a personalized prognostication model to determine eligibility. The first drug to be evaluated in MAGNET is lithium carbonate in *UNC13A* homozygous patients. MND-SMART is initially testing two IMPs (trazodone and memantine) against a single placebo arm with a seamless adaptive Phase II/Phase III multi-arm, multi-stage design, incorporating measures to maximize accessibility to pwALS including using broad inclusion criteria, liquid IMP and video/telephone assessments.

## Conclusion

Phenotypic heterogeneity, disease-related disability and the lack of sensitive and reliable outcome measures are some of the challenges in designing and conducting amyotrophic lateral sclerosis clinical trials. With improved understanding of amyotrophic lateral sclerosis disease biology and increasing number of promising candidate medicines being identified for evaluation, innovative trial designs can be instrumental in making trials more efficient, flexible, scalable and accessible. Together these advances will bring closer a new default where every pwALS has the opportunity to participate in a clinical trial.

## Supplementary material

Supplementary material is available at *Brain Communications* online.

## Supplementary Material

fcab242_Supplementary_Data

## Abbreviations

ALSFRS-R =: Amyotrophic Lateral Sclerosis Functional Rating Scale Revised
CAFS =: combined assessment of function and survival
CARE-MND =: Clinical Audit Research Evaluation for motor neurone disease
CTIMP =: Clinical Trial of Investigational Medicinal Product
EMA =: European Medicines Agency
EU =: European Union
EU CTR =: European Union Clinical Trial Registry
FDA =: Food and Drug Administration
HR =: hazard ratio
ICTRP =: International Clinical Trial Registry Platform
IMP =: investigational medicinal product
IQR =: interquartile range
MAMS =: multi-arm multi-stage
MND-SMART =: Motor Neurone Disease—Systematic Multi-Arm Adaptive Randomized Trial
PROACT =: Pooled Resources Open-Access Amyotrophic Lateral Sclerosis Clinical Trials
PRISMA =: Preferred Reporting Items for Systematic Reviews and Meta-Analyses
pwALS =: people with amyotrophic lateral sclerosis
RCT =: randomized controlled trial

## References

[1] Westeneng H-J , Debray TPA, Visser AE, et al Prognosis for patients with amyotrophic lateral sclerosis: Development and validation of a personalised prediction model. *Lancet Neurol*. 2018;17(5):423–433. 29598923 10.1016/S1474-4422(18)30089-9

[2] Dorst J , Ludolph AC, Huebers A. Disease-modifying and symptomatic treatment of amyotrophic lateral sclerosis. *Ther Adv Neurol Disord*. 2018;11:1756285617734734. 29399045 10.1177/1756285617734734 PMC5784546

[3] Miller RG , Mitchell JD, Moore DH, Cochrane Neuromuscular Group. Riluzole for amyotrophic lateral sclerosis (ALS)/motor neuron disease (MND). *Cochrane Database Syst Rev*. 2012;2012(3);Cd001447. 10.1002/14651858.CD001447.pub3 PMC7055506 22419278

[4] The Writing Group on behalf of the Edaravone (MCI-186) ALS 19 Study Group. Safety and efficacy of edaravone in well defined patients with amyotrophic lateral sclerosis: A randomised, double-blind, placebo-controlled trial. *Lancet Neurol*. 2017;16(7):505–512. 28522181 10.1016/S1474-4422(17)30115-1

[5] Paganoni S , Hendrix S, Dickson SP, et al Long-term survival of participants in the CENTAUR trial of sodium phenylbutyrate-taurursodiol in amyotrophic lateral sclerosis. *Muscle Nerve*. 2021;63(1):31–39. 33063909 10.1002/mus.27091 PMC7820979

[6] Paganoni S , Macklin EA, Hendrix S, et al Trial of sodium phenylbutyrate-taurursodiol for amyotrophic lateral sclerosis. *N Engl J Med*. 2020;383(10):919–930. 32877582 10.1056/NEJMoa1916945 PMC9134321

[7] Mora JS , Genge A, Chio A, et al; on behalf of the AB10015 STUDY GROUP. Masitinib as an add-on therapy to riluzole in patients with amyotrophic lateral sclerosis: A randomized clinical trial. *Amyotroph Lateral Scler Frontotemporal Degener*. 2020;21(1-2):5–14. 31280619 10.1080/21678421.2019.1632346

[8] Kaji R , Imai T, Iwasaki Y, et al Ultra-high-dose methylcobalamin in amyotrophic lateral sclerosis: A long-term phase II/III randomised controlled study. *J Neurol Neurosurg Psychiatry*. 2019;90(4):451–457. 30636701 10.1136/jnnp-2018-319294 PMC6581107

[9] Chiò A , Mazzini L, Mora G. Disease-modifying therapies in amyotrophic lateral sclerosis. *Neuropharmacology*. 2020;167:107986. 32062193 10.1016/j.neuropharm.2020.107986

[10] van Eijk RPA , Westeneng HJ, Nikolakopoulos S, et al Refining eligibility criteria for amyotrophic lateral sclerosis clinical trials. *Neurology*. 2019;92(5):e451–e460. 10.1212/WNL.0000000000006855 PMC6369899 30626653

[11] Unger JM , Vaidya R, Hershman DL, Minasian LM, Fleury ME. Systematic review and meta-analysis of the magnitude of structural, clinical, and physician and patient barriers to cancer clinical trial participation. *J Natl Cancer Inst*. 2019;111(3):245–255. 30856272 10.1093/jnci/djy221 PMC6410951

[12] NIH US Library of Medicine. ClinicalTrials.gov. https://clinicaltrials.gov. Accessed 9 April 2019.

[13] World Health Organisation. International Clinical Trials Registry Platform. http://apps.who.int/trialsearch/. Accessed 9 April 2019.

[14] European Medicines Agency. EU clinical trials register. https://www.clinicaltrialsregister.eu/. Accessed 9 April 2019.

[15] Aggarwal SP , Zinman L, Simpson E, et al; Northeast and Canadian Amyotrophic Lateral Sclerosis consortia. Safety and efficacy of lithium in combination with riluzole for treatment of amyotrophic lateral sclerosis: A randomised, double-blind, placebo-controlled trial. *Lancet Neurol*. 2010;9(5):481–488. 20363190 10.1016/S1474-4422(10)70068-5 PMC3071495

[16] Dupuis L , Dengler R, Heneka MT, et al; GERP ALS Study Group. A randomized, double blind, placebo-controlled trial of pioglitazone in combination with riluzole in amyotrophic lateral sclerosis. *PLoS One*. 2012;7(6):e37885. 22715372 10.1371/journal.pone.0037885 PMC3371007

[17] Meyer T , Maier A, Borisow N, et al Thalidomide causes sinus bradycardia in ALS. *J Neurol*. 2008;255(4):587–591. 18425621 10.1007/s00415-008-0756-3

[18] Kasper J , Heesen C, Kopke S, Fulcher G, Geiger F. Patients' and observers' perceptions of involvement differ. Validation study on inter-relating measures for shared decision making. *PLoS One*. 2011;6(10):e26255. 22043310 10.1371/journal.pone.0026255 PMC3197148

[19] European Medicines Agency Committee for Medicinal Products for Human Use. Withdrawal assessment report: Radicava. In: *European Medicines Agency Committee for Medicinal Products for Human Use, ed. EMA/CHMP/290284/2019*. Amsterdam; 2019.

[20] Abe K , Itoyama Y, Sobue G, et al; Edaravone ALS Study Group. Confirmatory double-blind, parallel-group, placebo-controlled study of efficacy and safety of edaravone (MCI-186) in amyotrophic lateral sclerosis patients. *Amyotroph Lateral Scler Frontotemporal Degener*. 2014;15(7-8):610–617. 25286015 10.3109/21678421.2014.959024 PMC4266079

[21] Exploratory double-blind, parallel-group, placebo-controlled study of edaravone (MCI-186) in amyotrophic lateral sclerosis (Japan ALS severity classification: Grade 3, requiring assistance for eating, excretion or ambulation). *Amyotroph Lateral Scler Frontotemporal Degener*. 2017;18 (Suppl 1):40–48. 28872915 10.1080/21678421.2017.1361441

[22] Okada M , Yamashita S, Ueyama H, Ishizaki M, Maeda Y, Ando Y. Long-term effects of edaravone on survival of patients with amyotrophic lateral sclerosis. *eNeurologicalSci*. 2018;11:11–14. 29928711 10.1016/j.ensci.2018.05.001 PMC6006910

[23] Lunetta C , Moglia C, Lizio A, et al; EDARAVALS Study Group. The Italian multicenter experience with edaravone in amyotrophic lateral sclerosis. *J Neurol*. 2020;267(11):3258–3267. 32556567 10.1007/s00415-020-09993-z

[24] Vu M , Tortorice K, Zacher J, et al Assessment of use and safety of edaravone for amyotrophic lateral sclerosis in the veterans affairs health care system. *JAMA Netw Open*. 2020;3(10):e2014645. 33017028 10.1001/jamanetworkopen.2020.14645 PMC7536587

[25] European Medicines Agency. Refusal of the marketing authorisation for Alsitek (masitinib). 2018.

[26] Amylyx. Amylyx Pharmaceuticals provides global regulatory update on AMX0035 for ALS. 2021. https://www.amylyx.com/2021/04/14/amylyx-pharmaceuticals-provides-global-regulatory-update-on-amx0035/. Accessed 27 July 2021.

[27] Smith R , Pioro E, Myers K, et al Enhanced bulbar function in amyotrophic lateral sclerosis: The Nuedexta treatment trial. *Neurotherapeutics*. 2017;14(3):762–772. 28070747 10.1007/s13311-016-0508-5 PMC5509619

[28] van den Berg LH , Sorenson E, Gronseth G, et al; Airlie House ALS Clinical Trials Guidelines Group. Revised Airlie House consensus guidelines for design and implementation of ALS clinical trials. *Neurology*. 2019;92(14):e1610–e1623. 30850440 10.1212/WNL.0000000000007242 PMC6448453

[29] Bedlack RS , Wicks P, Vaughan T, et al Lunasin does not slow ALS progression: Results of an open-label, single-center, hybrid-virtual 12-month trial. *Amyotroph Lateral Scler Frontotemporal Degener*. 2019;20(3-4):285–293. 30663902 10.1080/21678421.2018.1556698

[30] Cudkowicz ME , van den Berg LH, Shefner JM, et al; EMPOWER Investigators. Dexpramipexole versus placebo for patients with amyotrophic lateral sclerosis (EMPOWER): A randomised, double-blind, phase 3 trial. *Lancet Neurol*. 2013;12(11):1059–1067. 24067398 10.1016/S1474-4422(13)70221-7

[31] Meininger V , Genge A, van den Berg LH, et al; NOG112264 Study Group. Safety and efficacy of ozanezumab in patients with amyotrophic lateral sclerosis: A randomised, double-blind, placebo-controlled, phase 2 trial. *Lancet Neurol*. 2017;16(3):208–216. 28139349 10.1016/S1474-4422(16)30399-4

[32] Marin B , Boumédiene F, Logroscino G, et al Variation in worldwide incidence of amyotrophic lateral sclerosis: A meta-analysis. *Int J Epidemiol*. 2017;46(1):57–74. 27185810 10.1093/ije/dyw061 PMC5407171

[33] ClinicalTrials.gov. Identifier NCT02450552 clinical trial of ezogabine (retigabine) in ALS subjects. https://clinicaltrials.gov/ct2/show/NCT02450552. Accessed 3 February 2020.

[34] Shefner JM , Cudkowicz ME, Hardiman O, et al; VITALITY-ALS Study Group. A phase III trial of tirasemtiv as a potential treatment for amyotrophic lateral sclerosis. *Amyotroph Lateral Scler Frontotemporal Degener*. 2019;0(0):1–11. 31081694 10.1080/21678421.2019.1612922

[35] Mejzini R , Flynn LL, Pitout IL, Fletcher S, Wilton SD, Akkari PA. ALS genetics, mechanisms, and therapeutics: Where are we now? *Front Neurosci* . 2019;13:1310. 31866818 10.3389/fnins.2019.01310 PMC6909825

[36] Verber NS , Shepheard SR, Sassani M, et al Biomarkers in motor neuron disease: A state of the art review. *Front Neurol*. 2019;10:291. 31001186 10.3389/fneur.2019.00291 PMC6456669

[37] Donaghy C , Dick A, Hardiman O, Patterson V. Timeliness of diagnosis in motor neurone disease: A population-based study. *Ulster Med J*. 2008;77(1):18–21. 18269112 PMC2397016

[38] Leighton D , Newton J, Colville S, et al Clinical audit research and evaluation of motor neuron disease (CARE-MND): A national electronic platform for prospective, longitudinal monitoring of MND in Scotland. *Amyotrophic Lateral Scler Frontotemporal Degener*. 2019;20:242–250. 10.1080/21678421.2019.1582673 30889975

[39] Crockford C , Newton J, Lonergan K, et al ALS-specific cognitive and behavior changes associated with advancing disease stage in ALS. *Neurology*. 2018;91(15):e1370–e1380. 30209236 10.1212/WNL.0000000000006317 PMC6177274

[40] Radakovic R , Abrahams S. Multidimensional apathy: Evidence from neurodegenerative disease. *Curr Opin Behav Sci*. 2018;22:42–49.

[41] Beswick E , Park E, Wong C, et al A systematic review of neuropsychiatric and cognitive assessments used in clinical trials for amyotrophic lateral sclerosis [published online ahead of print, 2020 Sep 10]. *J Neurol.* 2020;10.1007/s00415-020-10203-z. doi:10.1007/s00415-020-10203-z PMC8563523 32910255

[42] Leighton DN , Parry J, Cleary D et al Phenotype-genotype characterisation of ‘long survivors’ with motor neurone disease in Scotland. *Amyotroph Lateral Scler Frontotemporal Degener*. 2018;19(supp1):63.

[43] Pupillo E , Messina P, Logroscino G, Beghi E, The SG, SLALOM Group. Long-term survival in amyotrophic lateral sclerosis: A population-based study. *Ann Neurol*. 2014;75(2):287–297. 24382602 10.1002/ana.24096

[44] Mateen FJ , Carone M, Sorenson EJ. Patients who survive 5 years or more with ALS in Olmsted County, 1925-2004. *J Neurol Neurosurg Psychiatry*. 2010;81(10):1144–1146. 20627966 10.1136/jnnp.2009.201251 PMC2946435

[45] Goyal NA , Berry JD, Windebank A, et al Addressing heterogeneity in ALS Clinical Trials. *Muscle Nerve*. 2020;62(2):156–166. 31899540 10.1002/mus.26801 PMC7496557

[46] Ludolph A , Drory V, Hardiman O, et al; WFN Research Group On ALS/MND. A revision of the El Escorial criteria - 2015. *Amyotrophic Lateral Scler Frontotemporal Degener*. 2015;16(5-6):291–292. 10.3109/21678421.2015.1049183 26121170

[47] Johnsen B , Pugdahl K, Fuglsang-Frederiksen A, et al Diagnostic criteria for amyotrophic lateral sclerosis: A multicentre study of inter-rater variation and sensitivity. *Clin Neurophysiol*. 2019;130(2):307–314. 30573424 10.1016/j.clinph.2018.11.021

[48] Shefner JM , Al-Chalabi A, Baker MR, et al A proposal for new diagnostic criteria for ALS. *Clin Neurophysiol*. 2020;131(8):1975–1978. 32387049 10.1016/j.clinph.2020.04.005

[49] Jayaprakash K , Glasmacher SA, Pang B, et al; CARE-MND Consortium. Riluzole prescribing, uptake and treatment discontinuation in people with amyotrophic lateral sclerosis in Scotland. *J Neurol*. 2020;267(8):2459–2461. 32447548 10.1007/s00415-020-09919-9 PMC7359150

[50] Newton J , Jayaprakash K, Glasmacher SA, et al Excellent reliability of the ALSFRS-R administered via videoconferencing: A study of people with motor neuron disease in Scotland. *J Neurol Sci*. 2020;416:116991. 32599295 10.1016/j.jns.2020.116991

[51] Franchignoni F , Mora G, Giordano A, Volanti P, Chiò A. Evidence of multidimensionality in the ALSFRS-R Scale: A critical appraisal on its measurement properties using Rasch analysis. *J Neurol Neurosurg Psychiatry*. 2013;84(12):1340–1345. 23516308 10.1136/jnnp-2012-304701

[52] Rooney J , Burke T, Vajda A, Heverin M, Hardiman O. What does the ALSFRS-R really measure? A longitudinal and survival analysis of functional dimension subscores in amyotrophic lateral sclerosis. *J Neurol Neurosurg Psychiatry*. 2017;88(5):381–385. 27888187 10.1136/jnnp-2016-314661

[53] van Eijk RPA , de Jongh AD, Nikolakopoulos S, et al An old friend who has overstayed their welcome: The ALSFRS-R total score as primary endpoint for ALS clinical trials. *Amyotrophic Lateral Scler Frontotemporal Degener*. 2021;22(3-4):300–307. 10.1080/21678421.2021.1879865 33527843

[54] Bedlack RS , Vaughan T, Wicks P, et al How common are ALS plateaus and reversals? *Neurology*. 2016;86(9):808–812. 26658909 10.1212/WNL.0000000000002251 PMC4793781

[55] Castrillo-Viguera C , Grasso DL, Simpson E, Shefner J, Cudkowicz ME. Clinical significance in the change of decline in ALSFRS-R. *Amyotroph Lateral Scler*. 2010;11(1-2):178–180. 19634063 10.3109/17482960903093710

[56] Atassi N , Berry J, Shui A, et al The PRO-ACT database: Design, initial analyses, and predictive features. *Neurology*. 2014;83(19):1719–1725. 25298304 10.1212/WNL.0000000000000951 PMC4239834

[57] de Jonge JC , Reinink H, Colam B, et al Regulatory delays in a multinational clinical stroke trial. *Eur Stroke J*. 2021;6(2):120–127. 34414286 10.1177/23969873211004845 PMC8370076

[58] Crow RA , Hart KA, McDermott MP, et al A checklist for clinical trials in rare disease: Obstacles and anticipatory actions—lessons learned from the FOR-DMD trial. *Trials*. 2018;19(1):291. 29793540 10.1186/s13063-018-2645-0 PMC5968578

[59] ALS Association. AMX0035 petition delivered to FDA. https://www.als.org/blog/amx0035-petition-delivered-fda. 2021. Accessed 27 July 2021.

[60] Feinberg BA , Gajra A, Zettler ME, Phillips TD, Phillips EG, Kish JK. Use of real-world evidence to support FDA approval of oncology drugs. *Value Health*. 2020;23(10):1358–1365. 33032780 10.1016/j.jval.2020.06.006

[61] The Lancet Neurology. Rapid drug access and scientific rigour: A delicate balance. *Lancet Neurol*. 2021;20(1):1. 33340471 10.1016/S1474-4422(20)30452-X

[62] Collins R , Bowman L, Landray M, Peto R. The magic of randomization versus the myth of real-world evidence. *N Engl J Med*. 2020;382(7):674–678. 32053307 10.1056/NEJMsb1901642

[63] OECD. Recommendation of the Council on the Governence of Clinical Trials. *OECD/LEGAL/0397*2021.

[64] Vesterinen HM , Connick P, Irvine CM, et al Drug repurposing: A systematic approach to evaluate candidate oral neuroprotective interventions for secondary progressive multiple sclerosis. *PLoS One*. 2015;10(4):e0117705. 25856304 10.1371/journal.pone.0117705 PMC4391783

[65] Dolmetsch R , Geschwind DH. The human brain in a dish: The promise of iPSC-derived neurons. *Cell*. 2011;145(6):831–834. 21663789 10.1016/j.cell.2011.05.034 PMC3691069

[66] Sandoe J , Eggan K. Opportunities and challenges of pluripotent stem cell neurodegenerative disease models. *Nat Neurosci*. 2013;16(7):780–789. 23799470 10.1038/nn.3425

[67] Miller T , Cudkowicz M, Shaw PJ, et al Phase 1–2 trial of antisense oligonucleotide tofersen for SOD1 ALS. *N Engl J Med*. 2020;383(2):109–119. 32640130 10.1056/NEJMoa2003715

[68] TRICALS. TRICALS Phase 3 MAGNET trial. https://www.tricals.org/trials/magnet/. Accessed 10 November 2020.

[69] Berry DA. Emerging innovations in clinical trial design. *Clin Pharmacol Ther*. 2016;99(1):82–91. 26561040 10.1002/cpt.285

[70] Pallmann P , Bedding AW, Choodari-Oskooei B, et al Adaptive designs in clinical trials: Why use them, and how to run and report them. *BMC Med*. 2018;16(1):29. 29490655 10.1186/s12916-018-1017-7 PMC5830330

[71] Kaufmann P , Thompson JL, Levy G, et al; QALS Study Group. Phase II trial of CoQ10 for ALS finds insufficient evidence to justify phase III. *Ann Neurol*. 2009;66(2):235–244. 19743457 10.1002/ana.21743 PMC2854625

[72] Piepers S , Veldink JH, de Jong SW, et al Randomized sequential trial of valproic acid in amyotrophic lateral sclerosis. *Ann Neurol*. 2009;66(2):227–234. 19743466 10.1002/ana.21620

[73] Verstraete E , Veldink JH, Huisman MH, et al Lithium lacks effect on survival in amyotrophic lateral sclerosis: A phase IIb randomised sequential trial. *J Neurol Neurosurg Psychiatry*. 2012;83(5):557–564. 22378918 10.1136/jnnp-2011-302021

[74] Cudkowicz ME , Titus S, Kearney M, et al Safety and efficacy of ceftriaxone for amyotrophic lateral sclerosis: A multi-stage, randomised, double-blind, placebo-controlled trial. *Lancet Neurol*. 2014;13(11):1083–1091. 25297012 10.1016/S1474-4422(14)70222-4 PMC4216315

[75] Angus DC , Alexander BM, Berry S, et al Adaptive platform trials: Definition, design, conduct and reporting considerations. *Nat Rev Drug Discov*. 2019;18(10):797–807. 31462747 10.1038/s41573-019-0034-3

[76] Parmar MK , Carpenter J, Sydes MR. More multiarm randomised trials of superiority are needed. *Lancet*. 2014;384(9940):283–284. 25066148 10.1016/S0140-6736(14)61122-3

[77] The RECOVERY Collaborative Group. Dexamethasone in hospitalized patients with Covid-19 — preliminary report. *N Engl J Med*. 2020;384:693–704. 32678530 10.1056/NEJMoa2021436 PMC7383595

[78] James ND , de Bono JS, Spears MR, et al Abiraterone for prostate cancer not previously treated with hormone therapy. *N Engl J Med*. 2017;377(4):338–351. 28578639 10.1056/NEJMoa1702900 PMC5533216

[79] James ND , Sydes MR, Clarke NW, et al STAMPEDE: systemic therapy for advancing or metastatic prostate cancer — a multi-arm multi-stage randomised controlled trial. *Clin Oncol*. 2008;20(8):577–581. 10.1016/j.clon.2008.07.002 18760574

[80] STAMPEDE. STAMPEDE: systemic therapy in advancing or metastatic prostate cancer: evaluation of drug efficacy. 2019.

[81] Berry SM , Petzold EA, Dull P, et al A response adaptive randomization platform trial for efficient evaluation of Ebola virus treatments: A model for pandemic response. *Clin Trials*. 2016;13(1):22–30. 26768569 10.1177/1740774515621721 PMC5583707

[82] Hague D , Townsend S, Masters L, et al; STAMPEDE and FOCUS4 investigators. Changing platforms without stopping the train: Experiences of data management and data management systems when adapting platform protocols by adding and closing comparisons. *Trials*. 2019;20(1):294. 31138292 10.1186/s13063-019-3322-7 PMC6540437

[83] Morrell L , Hordern J, Brown L, et al Mind the gap? The platform trial as a working environment. *Trials*. 2019;20(1):297. 31138284 10.1186/s13063-019-3377-5 PMC6540560

[84] Schiavone F , Bathia R, Letchemanan K, et al; past and present members of the STAMPEDE and FOCUS4 Trial Management Group. This is a platform alteration: A trial management perspective on the operational aspects of adaptive and platform and umbrella protocols. *Trials*. 2019;20(1):264. 31138317 10.1186/s13063-019-3216-8 PMC6540525

[85] Sydes MR , Parmar MK, James ND, et al Issues in applying multi-arm multi-stage methodology to a clinical trial in prostate cancer: The MRC STAMPEDE trial. *Trials*. 2009;10:39. 19519885 10.1186/1745-6215-10-39 PMC2704188

[86] Parmar MK , Sydes MR, Cafferty FH, et al Testing many treatments within a single protocol over 10 years at MRC Clinical Trials Unit at UCL: Multi-arm, multi-stage platform, umbrella and basket protocols. *Clin Trials*. 2017;14(5):451–461. 28830236 10.1177/1740774517725697 PMC5700799

[87] Wason J , Magirr D, Law M, Jaki T. Some recommendations for multi-arm multi-stage trials. *Stat Methods Med Res*. 2016;25(2):716–727. 23242385 10.1177/0962280212465498 PMC4843088

[88] Choodari-Oskooei B , Bratton DJ, Gannon MR, Meade AM, Sydes MR, Parmar MK. Adding new experimental arms to randomised clinical trials: Impact on error rates. *Clin Trials*. 2020;17(3):273–284. 32063029 10.1177/1740774520904346 PMC7263043

[89] Saville B , Quintana M, Broglio K, et al C8 The ALS platform trial: Design considerations and statistical efficiencies. *Amyotrophic Lateral Scler Frontotemporal Degener*. 2019;20:7.

[90] MND-SMART. MND-SMART Clinical trials for MND. www.mnd-smart.org. Accessed 3 February 2020.

[91] The ALS Association. Epidemiology of ALS and suspected clusters. http://www.alsa.org/als-care/resources/publications-videos/factsheets/epidemiology.html. Accessed 19 August 2019.

[92] The Scottish Motor Neuron Disease Register: A prospective study of adult onset motor neuron disease in Scotland. Methodology, demography and clinical features of incident cases in 1989. *J Neurol Neurosurg Psychiatry*. 1992;55(7):536–541. 1640227 10.1136/jnnp.55.7.536 PMC489161

[93] Worms PM. The epidemiology of motor neuron diseases: A review of recent studies. *J Neurol Sci*. 2001;191(1-2):3–9. 11676986 10.1016/s0022-510x(01)00630-x

[94] Alonso A , Logroscino G, Jick SS, Hernan MA. Incidence and lifetime risk of motor neuron disease in the United Kingdom: A population-based study. *Eur J Neurol*. 2009;16(6):745–751. 19475756 10.1111/j.1468-1331.2009.02586.x PMC3093130

