# Supplementary material for: Clinical trials in amyotrophic lateral sclerosis: a systematic review and perspective
Source: Brain Commun. 2021 Oct 23;3(4):fcab242. doi: 10.1093/braincomms/fcab242 (PMC8659356; doi:10.1093/braincomms/fcab242)
Supplement: fcab242_Supplementary_Data [file fcab242_supplementary_data.zip › Supplementary tables 27Nov2021.docx]

**Supplementary Tables**

Supplementary Table 1 Investigational Medicinal Products evaluated in Phase II, Phase II/III and Phase III CTIMPs registered, completed or published from 1 Jan 2008. *Involved in a Multiarm trial ^ used in combination in 1 trial § multiphase Phase I - III trial

Supplementary Table 2 IMPs grouped by route and form used.

Supplementary Table 3 Phase II, Phase II/III, Phase III CTIMPs assessing potential disease modifying drugs in ALS which recruited in (a) the United Kingdom and (b) the United States between 2008-April 2019 identified by search of trial registries and PubMed search.*UK and US participants based on published data when available; when not available, estimated using percentage of centres in the UK or US multiplied by total participants. Estimated new cases in year 2008-April 2019 based on data on from Office of National Statistics, and annual incidence of 2/100000^91-94^ UK: 14450 cases, US: 71061 cases. Trial participation percentage UK: 4.2%, US: 7.9%

Supplementary Table 1

| **IMP** | **Phase II** | **Phase II/III** | **Phase III** | **Total** |
| --- | --- | --- | --- | --- |
| Lithium | 6 | 2 | 2 | **10** |
| Edaravone (MCI-186) | 1 | 1 | 3 | **5** |
| Tirasemtiv (CK-2017357) | 4 |  | 1 | **5** |
| Arimoclomol | 2 | 1 | 1 | **4** |
| Granulocyte Colony Stimulating Factor | 3 | 1 |  | **4** |
| Creatine*^ | 3 |  |  | **3** |
| ILB | 3 |  |  | **3** |
| Memantine | 2 | 1 |  | **3** |
| Mexiletine | 3 |  |  | **3** |
| Rasagiline | 3 |  |  | **3** |
| Acthar | 2 |  |  | **2** |
| Biotin (MD1003) | 2 |  |  | **2** |
| Ceftriaxone§ | 1 |  | 1 | **2** |
| Deferiprone | 1 | 1 |  | **2** |
| Dexpramipexole (KNS-760704) | 1 |  | 1 | **2** |
| Erythropoietin | 1 |  | 1 | **2** |
| Ezogabine (Retigabine) | 2 |  |  | **2** |
| Fasudil | 2 |  |  | **2** |
| IL-2 | 2 |  |  | **2** |
| Levosimendan (ODM-109) | 1 |  | 1 | **2** |
| Masitinib |  | 1 | 1 | **2** |
| NP001 | 2 |  |  | **2** |
| Pimozide | 2 |  |  | **2** |
| Riluzole oral soluble film | 2 |  |  | **2** |
| RNS60 | 2 |  |  | **2** |
| Talampanel | 2 |  |  | **2** |
| Tauroursodeoxycholic acid (TUDCA) | 1 |  | 1 | **2** |
| Thalidomide | 2 |  |  | **2** |
| Ursodeoxycholic acid | 1 |  | 1 | **2** |
| 3,4 Diaminopyridine | 1 |  |  | **1** |
| Acetyl-l-carnitine | 1 |  |  | **1** |
| AMX0035 (Sodium phenylbutyrate and tauroursodeoxycholic acid) | 1 |  |  | **1** |
| Anakinra | 1 |  |  | **1** |
| Basiliximab\|Methylprednisolone\|Prednisone\|Tacrolimus\| Mycophenolate mofetil | 1 |  |  | **1** |
| BIIB067 (IONIS SOD1Rx) |  |  | 1 | **1** |
| Bromocriptine (NDDPX-08) | 1 |  |  | **1** |
| CBD |  |  | 1 | **1** |
| Celecoxib* | 1 |  |  | **1** |
| Cistanche Total Glycosides | 1 |  |  | **1** |
| CK-2127107 (Reldesemtiv) | 1 |  |  | **1** |
| Coenzyme Q10 | 1 |  |  | **1** |
| Colchicine | 1 |  |  | **1** |
| Dimethyl fumarate (Tecfidera) | 1 |  |  | **1** |
| EPI-589 | 1 |  |  | **1** |
| Erythropoeitin | 1 |  |  | **1** |
| Fingolimod (Gilenya) | 1 |  |  | **1** |
| Flecainide | 1 |  |  | **1** |
| Fycompa (Perampanel) | 1 |  |  | **1** |
| Glatiramer acetate | 1 |  |  | **1** |
| GM604 | 1 |  |  | **1** |
| Gold Nanocrystals | 1 |  |  | **1** |
| Guanabenz | 1 |  |  | **1** |
| Ibudilast (MN-166) | 1 |  |  | **1** |
| Inosine | 1 |  |  | **1** |
| Insulin like growth factor type 1 |  |  | 1 | **1** |
| L-Serine | 1 |  |  | **1** |
| Lunasin | 1 |  |  | **1** |
| Mecasin | 1 |  |  | **1** |
| Methylcobalamin |  |  | 1 | **1** |
| Minocycline* | 1 |  |  | **1** |
| Nanocurcumin | 1 |  |  | **1** |
| Nuedexta (dextromethorphan/quinidine) | 1 |  |  | **1** |
| Olesoxime (TRO19622) |  |  | 1 | **1** |
| Ozanezumab (GSK1223249) | 1 |  |  | **1** |
| Penicillin G and hydrocortisone | 1 |  |  | **1** |
| Perampanel | 1 |  |  | **1** |
| Pioglitazone | 1 |  |  | **1** |
| Ranolazine | 1 |  |  | **1** |
| Rapamycin | 1 |  |  | **1** |
| SB-509 | 1 |  |  | **1** |
| Sodium phenylbutyrate | 1 |  |  | **1** |
| Sodium Valproate |  |  | 1 | **1** |
| Tamoxifen | 1 |  |  | **1** |
| Tocilizumab | 1 |  |  | **1** |
| Triumeq | 1 |  |  | **1** |
| YAM80 | 1 |  |  | **1** |

Supplementary Table 2

| **Route/Form of IMP** | **Number of trials** |
| --- | --- |
| Oral, form not specified | 13 |
| Oral tablet | 38 |
| Oral capsule | 25 |
| Oral powder | 2 |
| Oral soluble | 3 |
| Oral capsules and powder | 2 |
| Oral liquid | 2 |
| Subcutaneous | 14 |
| Intravenous | 17 |
| Intramuscular | 2 |
| Intravenous and oral | 1 |
| Intrathecal | 1 |
| Nebulised | 1 |
| Intravenous and nebulised | 1 |
| Intravenous and subcutaneous | 1 |
| Not specified | 2 |

Supplementary Table 3

| 1. **Trial participation in United Kingdom** | | | | | | |
| --- | --- | --- | --- | --- | --- | --- |
| **Trial ID** | **Total participants** | **UK participants** | **UK centres** | **Total centres** | **UK centres percentage** | **UK participants*** |
| NCT00326625 | 366 | 56 | 1 | 6 | 17 | **56** |
| ISRCTN 83178718 | 214 | 214 | 10 | 10 | 100 | **214** |
| NCT00868166 | 512 | 52 | 2 | 15 | 13 | **52** |
| NCT01281189 | 943 | NA | 6 | 82 | 7 | **69** |
| NCT01753076 | 304 | 21 | 3 | 35 | 9 | **21** |
| NCT01709149 | 711 | 50 | 6 | 75 | 8 | **50** |
| NCT02487407 | 66 | 25 | 5 | 11 | 45 | **25** |
| NCT02623699 | 244 | NA | 1 | 15 | 7 | **16** |
| NCT02496767 | 743 | 7 | 4 | 81 | 5 | **7** |
| NCT03039673 | 216 | 72 | 7 | 17 | 41 | **72** |
| NCT03491462 | 231 | NA | 1 | 32 | 3 | **7** |
| NCT03505021 | 450 | NA | 3 | 99 | 3 | **14** |
|  |  |  |  |  |  |  |
| **TOTAL** | 5000 | 497 |  |  |  | **603** |
|  |  |  |  |  |  |  |
| **(b) Trial participation in United States** | | | | | | |
| **Trial ID** | **Total participants** | **Published US participants** | **US centres** | **Total centres** | **US centres percentage** | **US participants*** |
| NCT00035815 | 330 | 330 | 19 | 20 | 95 | **330** |
| NCT00070993 | 110 | 107 | 6 | 6 | 100 | **107** |
| NCT00107770 | 40 | 40 | 10 | 10 | 100 | **40** |
| NCT00140452 | 24 | 23 | 1 | 1 | 100 | **23** |
| NCT00243932 | 185 | 185 | 19 | 19 | 100 | **185** |
| NCT00349622 | 513 | 444 | 50 | 58 | 86 | **444** |
| NCT00355576 | 86 | 86 | 19 | 19 | 100 | **86** |
| NCT00647296 | 102 | 102 | 21 | 21 | 100 | **102** |
| NCT00696332 | 559 | NA | 7 | 24 | 29 | **163** |
| NCT00706147 | 38 | 38 | 2 | 2 | 100 | **38** |
| NCT00748501 | 45 | NA | 6 | 6 | 100 | **45** |
| NCT00790582 | 100 | 100 | 10 | 10 | 100 | **100** |
| NCT00818389 | 84 | NA | 22 | 37 | 60 | **50** |
| NCT01089010 | 67 | 67 | 15 | 15 | 100 | **67** |
| NCT01232738 | 36 | 33 | 9 | 10 | 90 | **33** |
| NCT01257581 | 60 | 60 | 9 | 9 | 100 | **60** |
| NCT01281189 | 943 | NA | 43 | 82 | 52 | **495** |
| NCT01281631 | 136 | 136 | 17 | 17 | 100 | **136** |
| NCT01378676 | 50 | 49 | 9 | 9 | 100 | **49** |
| NCT01486849 | 28 | NA | 11 | 11 | 100 | **28** |
| NCT01709149 | 711 | NA | 44 | 75 | 59 | **417** |
| NCT01753076 | 304 | NA | 4 | 35 | 11 | **35** |
| NCT01786174 | 30 | 30 | 4 | 4 | 100 | **30** |
| NCT01786603 | 80 | 80 | 10 | 10 | 100 | **80** |
| NCT01806857 | 90 | 90 | 7 | 7 | 100 | **90** |
| NCT01849770 | 75 | 75 | 10 | 10 | 100 | **75** |
| NCT01854294 | 12 | 12 | 2 | 2 | 100 | **12** |
| NCT01884571 | 31 | 31 | 3 | 3 | 100 | **31** |
| NCT01906658 | 43 | 43 | 17 | 17 | 100 | **43** |
| NCT02118727 | 90 | NA | 11 | 11 | 100 | **90** |
| NCT02238626 | 71 | NA | 1 | 1 | 100 | **71** |
| NCT02450552 | 65 | NA | 12 | 12 | 100 | **65** |
| NCT02460679 | 20 | NA | 3 | 3 | 100 | **20** |
| NCT02469896 | 22 | NA | 5 | 5 | 100 | **22** |
| NCT02496767 | 743 | NA | 49 | 81 | 61 | **449** |
| NCT02623699 | 144 | NA | 9 | 17 | 53 | **76** |
| NCT02709330 | 60 | 60 | 1 | 1 | 100 | **60** |
| NCT02781454 | 60 | NA | 10 | 10 | 100 | **60** |
| NCT02794857 | 138 | NA | 21 | 22 | 96 | **132** |
| NCT03068754 | 213 | NA | 39 | 69 | 57 | **120** |
| NCT03127514 | 132 | NA | 25 | 25 | 100 | **132** |
| NCT03160898 | 458 | NA | 47 | 64 | 73 | **336** |
| NCT03168711 | 30 | NA | 3 | 3 | 100 | **30** |
| NCT03456882 | 142 | NA | 1 | 20 | 5 | **7** |
| NCT03472950 | 20 | NA | 1 | 1 | 100 | **20** |
| NCT03491462 | 231 | NA | 10 | 29 | 35 | **80** |
| NCT03505021 | 450 | NA | 48 | 104 | 46 | **208** |
| NCT03580616 | 50 | NA | 1 | 1 | 100 | **50** |
| NCT03679975 | 9 | NA | 1 | 1 | 100 | **9** |
| PMID:19961264 | 59 | 59 | 2 | 2 | 100 | **59** |
| PMID:21321491 | 17 | 17 | 1 | 1 | 100 | **17** |
| NCT00244244 | 84 | 84 | 10 | 10 | 100 | **84** |
| PMID:20839903 | 20 | 20 | 1 | 1 | 100 | **20** |
| PMID:19935406 | 18 | 18 | 1 | 1 | 100 | **18** |
|  |  |  |  |  |  |  |
| **Total number of participants** |  |  |  |  |  | **5629** |
